# The impacts of natural product miltirone and the CYP2D6 pharmacogenetic phenotype on fluoxetine metabolism

**DOI:** 10.3389/fphar.2024.1373048

**Published:** 2024-04-29

**Authors:** Xiaodan Zhang, Qingqing Li, Xinwu Ye, Qing Chen, Chen Chen, Guoxin Hu, Likang Zhang, Lianguo Chen

**Affiliations:** ^1^ Wenzhou Seventh People’s Hospital, Wenzhou, Zhejiang, China; ^2^ Department of Clinical Pharmacy, The First Affiliated Hospital of Wenzhou Medical University, Wenzhou, Zhejiang, China; ^3^ Renji College, Wenzhou Medical University, Wenzhou, Zhejiang, China

**Keywords:** CYP2D6 variants, fluoxetine, norfluoxetine, miltirone, drug‒drug interaction

## Abstract

**Introduction: **To study the effects of drug-induced CYP2D6 activity inhibition and genetic polymorphisms on fluoxetine metabolism, rat liver microsomes (RLMs) and SD rats were used to investigate the potential drug‒drug interactions (DDIs), and CYP2D6 http://muchong.com/t-10728934-1 recombinant baculosomes were prepared and subjected to catalytic reactivity studies.

**Methods and Results:** All analytes were detected by ultraperformance liquid chromatography–tandem mass spectrometry (UPLC‒MS/MS). After screening for 27 targeted natural products, miltirone was identified as having obvious inhibitory effect on fluoxetine metabolism in RLMs. *In vivo*, the concentration of fluoxetine in rat blood increased markedly after miltirone administration. The molecular docking results showed that miltirone bound more strongly to CYP2D6 than fluoxetine, and PHE120 may be the key residue leading to the inhibition of CYP2D6-mediated fluoxetine N-demethylation by miltirone. In terms of the genetic polymorphism of CYP2D6 on fluoxetine metabolism, the intrinsic clearance values of most variants were significantly altered. Among these variants, CYP2D6*92 and CYP2D6*96/Q424X were found to be catalytically inactive for fluoxetine metabolism, five variants (CYP2D6*89/L142S, *97/F457L, *R497, *V342M and *R344Q) exhibited markedly increased clearance values (>125.07%) and seven variants (CYP2D6*2, *10, *87/A5V, *93/T249P, *E215K, *R25Q and *R440C) exhibited significantly decreased clearance values (from 6.62% to 66.79%) compared to those of the wild-type.

**Conclusion:** Our results suggest that more attention should be given to subjects in the clinic who take fluoxetine and also carry one of these infrequent CYP2D6 alleles or are coadministered drugs containing miltirone.

## 1 Introduction

Fluoxetine (FLX), a selective serotonin reuptake inhibitor (SSRI), has been used to treat depression, anxiety and even premature ejaculation (Kryst et al., 2022; Liu et al., 2022; Zhou et al., 2021). Compared to tricyclic antidepressants or monoamine oxidase inhibitors, SSRIs are less toxic, but there are still articles reporting that they can inhibit Na^+^, Ca^2+^, and K^+^ channels during treatment (Bruggeman et al., 2023). Moreover, it was noted that the toxicity of fluoxetine was related to atrial fibrillation and bradycardia (Bruggeman and O'Day, 2023), even a case of death (Sallee et al., 2000).

To improve quality of life and alleviate potential side effects, combining herbal and conventional medicines is common in East Asia. However, this combination approach poses the potential risks of adverse drug‒drug interactions (DDIs) (Jiang et al., 2022). From a molecular mechanism perspective, inhibiting metabolic enzyme activity is a key factor in the occurrence of adverse drug interactions. Some studies have suggested that CYP2C9, CYP2C19 and especially CYP2D6 play key roles in fluoxetine N-demethylation (Fang et al., 2017; Mandrioli et al., 2006; Margolis et al., 2000; von Moltke et al., 1997). Therefore, we investigated which natural compounds may interact with fluoxetine and elucidated their inhibitory mechanisms *in vivo* and *in vitro*.

Interestingly, the CYP enzyme system exhibits not only inhibitory potential but also genetic polymorphisms. Variations in enzymatic activity resulting from genetic polymorphisms can lead to wide variations in metabolic rates. To date, our laboratory has reported on the *in vitro* effects of 38 CYP2C9 variants and 30 CYP2C19 variants on fluoxetine metabolism, and most CYP2C9 and CYP2C19 isoforms exhibited notably lower clearance than did the corresponding wild-type for the N-demethylation of fiuoxetine, which should be receive increased attention from doctors when prescribing fluoxetine to patients who are poor metabolizers (Ji et al., 2015; Fang et al., 2017). Like other P450 subfamily members, the CYP2D6 gene shows a high rate of interindividual variation among different races and impacts drug responses to many commonly dispensed drugs, including opioids and antidepressants (Kehinde et al., 2023). To date, we have identified 22 novel CYP2D6 variants associated with different enzymatic functions and activities (Qian et al., 2013). To our knowledge, the effect of CYP2D6 polymorphism on fluoxetine metabolism has not been reported. Therefore, the enzymatic activities of these CYP2D6 variants toward fluoxetine were characterized in insect microsomes expressing wild-type and variant CYP2D6 according to a previously reported method (Ye et al., 2022).

## 2 Materials and methods

### 2.1 Chemical reagents and materials

Fluoxetine hydrochloride, norfluoxetine (NFLX) and formic acid were purchased from Sigma‒Aldrich (St. Louis, MO, United States). Twenty-seven natural products were obtained from Shanghai Chuangsai Technology Co., Ltd. (Shanghai, China). Paroxetine hydrochloride, as an internal standard (IS), was purchased from Toronto Research Chemicals, Inc. (Toronto, ON, Canada). sf21 insect microsomes expressing wild-type and variant CYP2D6, cytochrome b5 and reductase were obtained from Beijing Hospital (Beijing, China). NADPH was obtained from Roche Pharmaceuticals Ltd. (Basel, Switzerland), and the NADPH regeneration system was obtained from Promega (Madison, WI, United States). Rat liver microsomes (RLMs) were obtained from Corning Life Sciences Co., Ltd. The organic solvents used were purchased from Merck (Darmstadt, Germany).

### 2.2 Enzymatic studies

To research the possible DDIs of fluoxetine, 27 natural products were screened with the incubation assay of RLMs. The reactions were set up as follows. RLMs (0.3 mg/mL), NADPH (1 mM), fluoxetine (10 μM) and one of 27 natural products (100 μM) were mixed in Tris-HCl (100 μM) in a final volume of 200 μL. The concentration of fluoxetine was selected based on the K_m_ value reported previously (Ring et al., 2001). After preincubation for 5 min at 37 °C, the reaction was started by the addition of the NADPH regeneration system (3.3 mmol/L glucose 6-phosphate, 3.3 mmol/L MgCl_2_, 1.3 mmol/L NADP^+^ and 0.4 units/mL glucose-6-phosphate dehydrogenase) and stopped after 30 min with the addition of precooled acetonitrile (400 μL) containing IS (0.5 μg/mL). After vortexing for 2 min, the mixture was centrifuged at 13,000 rpm for 10 min. The supernatant (100 μL) was mixed with 200 μL of ddH_2_O, and an aliquot of the mixture (3 μL) was analyzed by ultraperformance liquid chromatography–tandem mass spectrometry (UPLC‒MS/MS). The relative enzymatic activity was measured after the formation of norfluoxetine.

According to the screening results, the effect of miltirone on the metabolic parameters of fluoxetine in RLMs was studied. Different concentrations of miltirone (0, 0.01, 0.1, 1, 10, 25, 50 and 100 μM) were mixed into the reaction system as mentioned above. Then, these reactions were started and stopped following the abovementioned steps, and the products were analyzed by UPLC‒MS/MS.

To investigate whether miltirone has the potential for causing time-dependent CYP inhibition, IC_50_ shift experiments were further carried out (Bao et al., 2018; Dong et al., 2021; Wang et al., 2020). RLMs (0.3 mg/mL) and different concentrations of miltirone were mixed and preincubated with or without 1 mM NADPH in Tris-HCl (100 μM) in a final volume of 200 μL at 37 °C for 30 min to identify weak inactivation. Then, fluoxetine (10 µM) was added to the mixture for another 30 min of incubation, and the supernatant was analyzed by UPLC‒MS/MS.

### 2.3 Molecular docking study

Molecular docking analysis was performed to confirm the drug binding conformations of miltirone to human CYP2C9 (PDB code 1R9O), CYP2C19 (PDB code 4KFO) and CYP2D6 (PDB code 4WNW). The ligands and proteins needed for molecular docking were prepared and docked with the software programs AutoDock Vina and PyRx software programs. Discovery Studio 2020 Client was used for visual analysis.

### 2.4 Pharmacokinetics research in rats

Sprague‒Dawley rats (male, 200 ± 10 g) were obtained from the Experimental Animal Research Center of Wenzhou Medical University (Wenzhou, China) and housed at 25 °C under a natural light-dark cycle. The animal experiments were approved by the Animal Ethics Committee of Wenzhou Medical University (wydw 2023-0461). All rats were randomly divided into 2 groups (6 in each group): the control group and the miltirone (coadministration) group. Miltirone and fluoxetine were dissolved in 5% Tween and vegetable oil, respectively. The rats in the miltirone group were orally administered miltirone (40 mg/kg), while those in the control group were given 5% Tween in the same manner for comparison. After 0.5 h, fluoxetine (8 mg/kg) was administered to all rats by gavage. Blood samples (approximately 300 μL) were collected via the caudal vein and placed in heparinized 1.5 mL tubes at 0.5, 1, 2, 3, 4, 6, 8, 12, 24 and 48 h after fluoxetine administration. Adequate amounts of urine were obtained by directly catching rats to collect urine at 12, 24 and 48 h. These samples were centrifuged (10,000 × *g*) immediately for 10 min after collection. Then, 100 μL of each plasma or urine sample was stored at −80 °C before analysis.

### 2.5 Enzymatic studies of fluoxetine with CYP2D6 variants

Wild-type CYP2D6 and 24 CYP2D6 variants (2 common variants and 22 defective allelic variants) were used for the enzymatic studies. The incubation procedures (60 min) were as follows. First, equal amounts of the P450 from insect microsomes (5–20 pmol), cytochrome b5 (5–20 pmol) and fluoxetine (10–300 μM) were mixed in potassium phosphate buffer in a final volume of 200 μL (100 mM, pH 7.4). These reactions were then started and stopped following the abovementioned steps. The supernatants were analyzed by UPLC‒MS/MS. The experiments were performed in triplicate.

### 2.6 Equipment and UPLC‒MS/MS analysis

The levels of fluoxetine, norfluoxetine and paroxetine were determined by a Waters ACQUITY UPLC‒MS/MS system (Waters, Milford, MA, United States) according to our previous articles (Ji et al., 2015; Fang et al., 2017).

### 2.7 Statistical analysis

The Michaelis‒Menten equation or the substrate inhibition equation was used to analyze and obtain the kinetic parameters (K_m_ and V_max_) after incubation, with calculations performed in GraphPad Prism 5 software (San Diego, CA, United States). One-way analysis was used to analyze the *in vitro* variances. Noncompartmental analysis was used for the analyte pharmacokinetic parameters of analytes, including the area under the time-concentration curve (AUC), half-life (T_1/2_), the maximum of blood concentration (C_max_), blood clearance rate (CL), apparent volume of distribution (V) and mean retention time (MRT), *in vivo* with DAS 3.0 software. The mean values in the miltirone and control groups were analyzed by *t* tests.

## 3 Results

### 3.1 Fluoxetine metabolism was inhibited by miltirone *in vitro*

**FIGURE 1:**
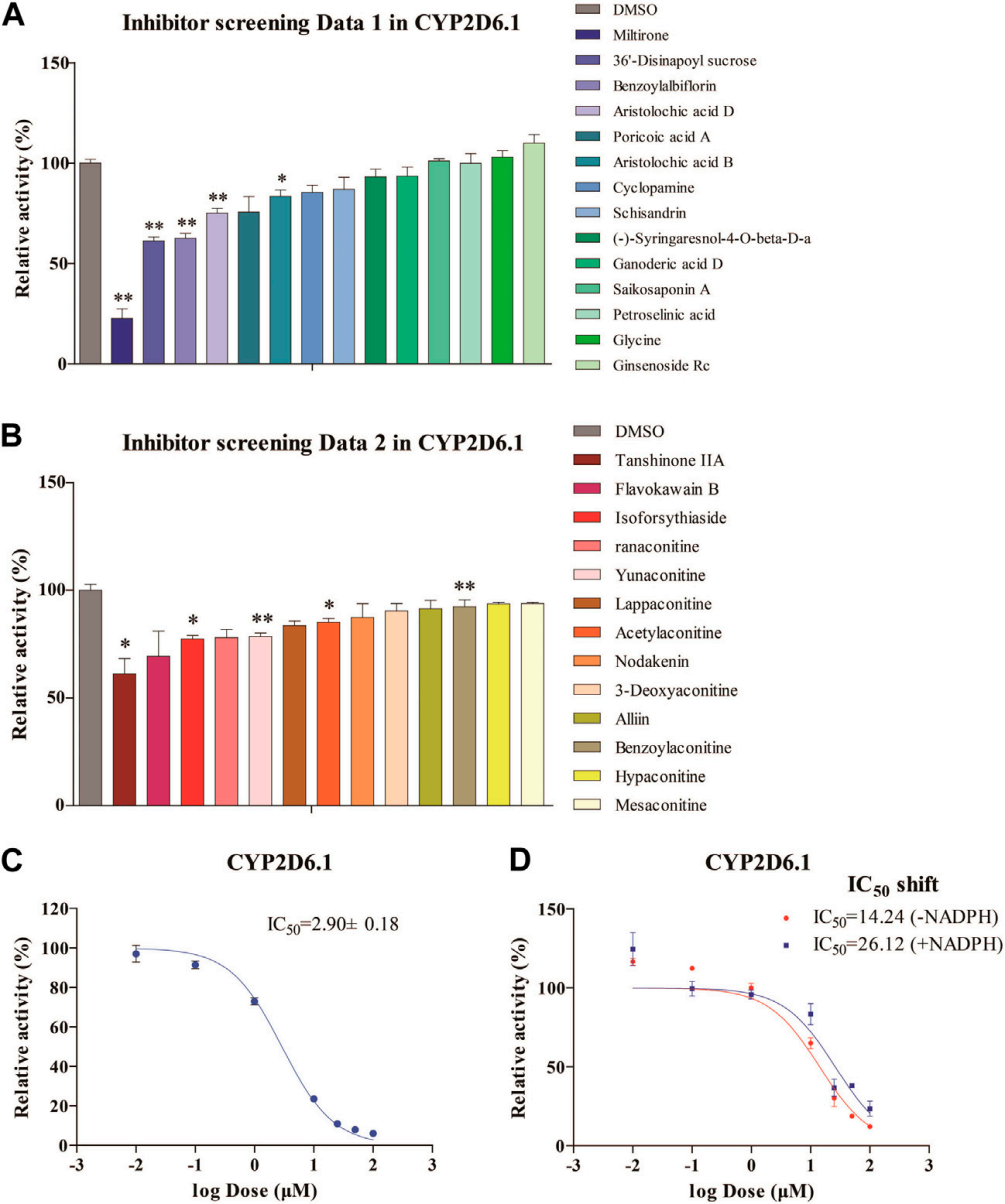
Enzymatic studies of CYP2D6*1 with inhibitors **(A,B)**, enzymatic studies with miltirone in RLMs **(C)**, and IC_50_ shift assays with miltirone in RLMs **(D)**. *p < 0.05 and **p < 0.01 versus the control group.

The inhibitory effects of 27 natural products on RLMs are summarized in Figures 1A, B. These products exhibited different inhibitory effects on CYP activity *in vitro*. Among these compounds, miltirone had the greatest inhibitory effect. Moreover, our results showed that the CYP inhibitions by miltirone was greater than that of tanshinone IIA, which was consistent with the results of a previous study (Zhou et al., 2013). The miltirone inhibition of RLMs data are summarized in Figure 1C, which gave an IC_50_ value of 2.9 μM. The results of the IC_50_ shift assays in Figure 1D show that the IC_50_ values were 26.12 and 14.24 μM after preincubation with and without NADPH, respectively.

### 3.2 Molecular docking study

**FIGURE 2:**
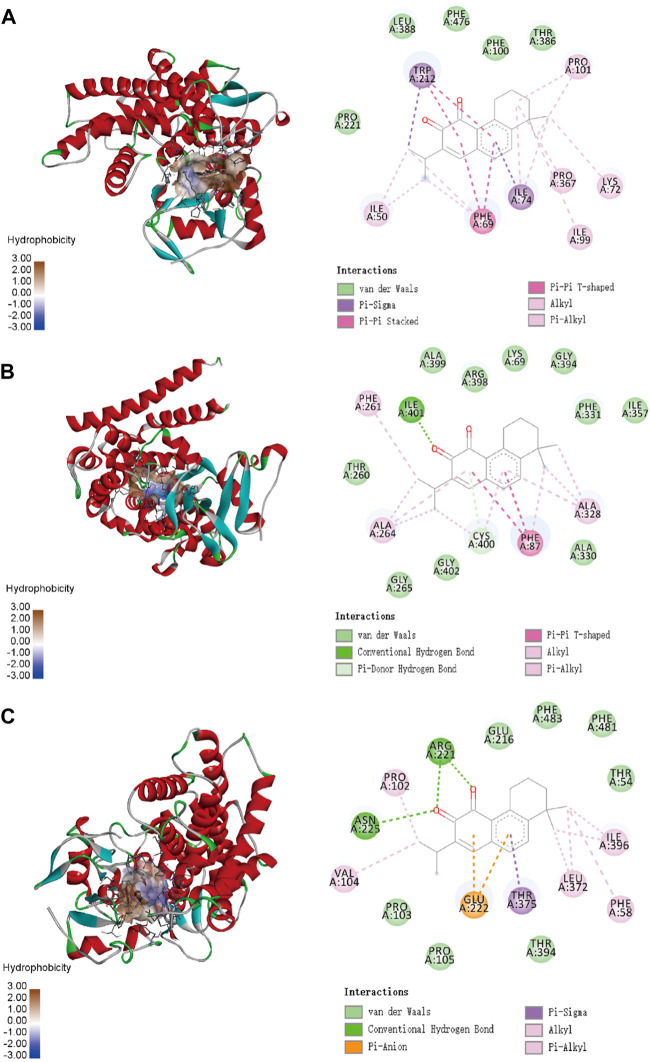
Molecular docking analysis illustrating the favorable binding positions of miltirone in the active cavity of human CYP2C9 **(A)**, CYP2C19 **(B)** and CYP2D6 **(C)**.

**FIGURE 3:**
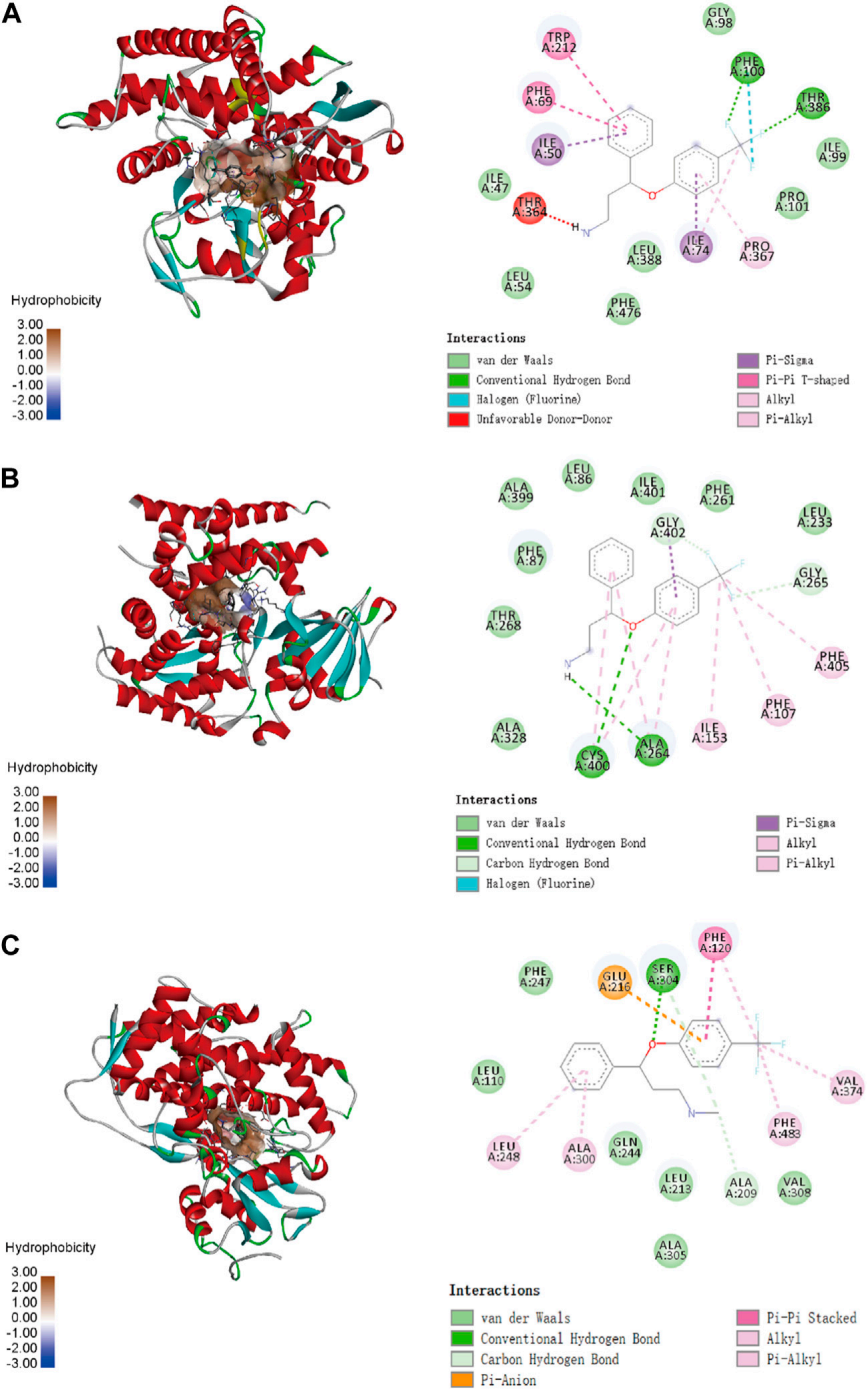
Molecular docking analysis illustrating the favorable binding positions of fluoxetine in the active cavity of human CYP2C9 **(A)**, CYP2C19 **(B)** and CYP2D6 **(C)**.

To confirm the ligand binding conformations of miltirone in the active sites of certain human CYP450 isoforms, a molecular docking study was performed. The free binding energies indicated that miltirone had stronger binding affinity than did fluoxetine (Table 1). As shown in Figure 2A, molecular docking analysis revealed that three key residues (ILE50, PHE69 and PHE212) were critical for the binding of miltirone and fluoxetine. Rings B and C of miltirone have strong pi-alkyl interactions with ILE50, PHE 69 and PHE 212. PHE 69 and PHE 212 also interact with fluoxetine through pi-pi T-shaped interactions. Moreover, ILE50 participated in a sigma–pi interaction with fluoxetine (Figure 3A). These interactions might be responsible for the inhibitory effect of miltirone on CYP2C9-mediated fluoxetine N-demethylation. As shown in Figure 2B, miltirone interacts with the side chains of ALA264 and CYS400 via alkyl interactions. Furthermore, miltirone also binds to CYS400 via pi–donor hydrogen bonding or pi–sulfur interactions. However, fluoxetine participates in an alkyl–pi interaction and/or conventional hydrogen bond with ALA264 and CYS400 (Figure 3B). These results suggested that miltirone could bind to the fluoxetine binding site on human CYP2C9. As shown in Figure 2C and Figure 3C, miltirone and fluoxetine do not share the same binding site in the crystal structure of CYP2D6. However, a previous article reported that the type of interaction between miltirone and CYP2D6 is a pi–pi interaction with PHE120 in another crystal structure of CYP2D6 (PDB code 3QM4), which is located in the fluoxetine binding site in our docking study (Zhou et al., 2013). Thus, PHE120 may be a key residue leading to the inhibition of CYP2D6-mediated fluoxetine N-demethylation by miltirone.

**TABLE 1:** Free binding energies (in kcal/mol) of miltirone and fluoxetine docked into the active cavities of human CYP2C9, CYP2C19, and CYP2D6, respectively.

CYP isoforms	Miltirone	Fluoxetine
CYP2C9	−9.9 kcal/mol	−8.1 kcal/mol
CYP2C19	−9.3 kcal/mol	−7.9 kcal/mol
CYP2D6	−9.2 kcal/mol	−8.2 kcal/mol

### 3.3 Miltirone inhibited the inhibited by miltirone *in vivo*

**FIGURE 4:**
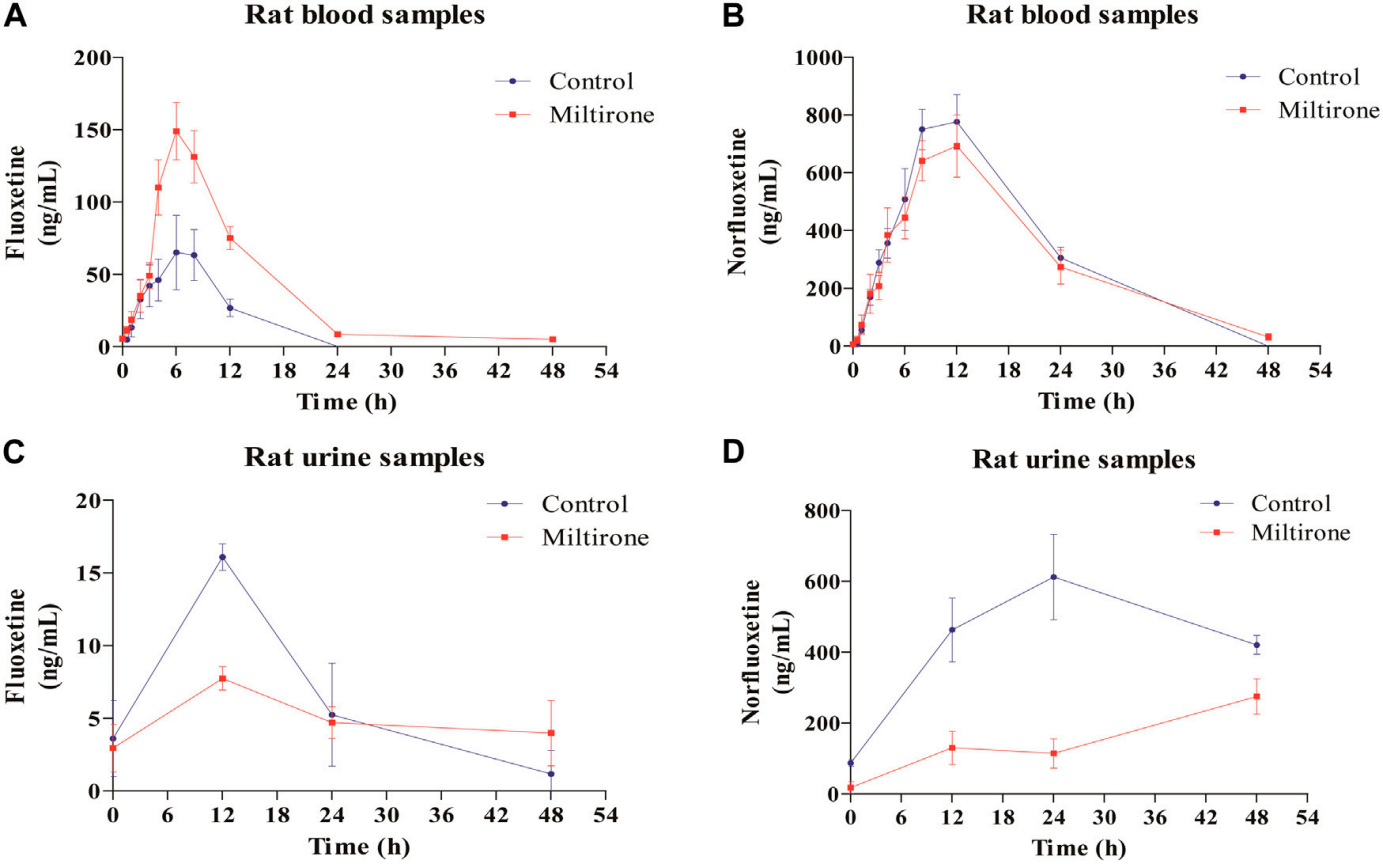
Mean concentration-time curves of fluoxetine **(A)** and norfluoxetine **(B)** in blood, and of fluoxetine **(C)** and norfluoxetine **(D)** in urine in the two groups (n = 6).

As shown in Figure 4, the plasma concentration of fluoxetine decreased with miltirone exposure after comedication. The AUC_(0–t)_, T_1/2z_ and CLz of fluoxetine, which are summarized in Table 2, were significantly different between the control and miltirone groups. The CLz of fluoxetine in the coadministration group decreased by 69.6% (*p* < 0.05) compared with that in the control group. Moreover, the miltirone group exhibited significantly greater AUC_(0–t)_ (2.6-fold) and T_1/2z_ (2.4- fold) values. In addition, the C_max_ of fluoxetine increased by 96.1% (*p* < 0.05) in the miltirone group. As shown in Table 3, there were no significant differences in the pharmacokinetic parameters of norfluoxetine between the two groups (*p* > 0.05). However, the levels of norfluoxetine in the urine at different times after miltirone administration were lower than those in the control group, which was different from the findings for fluoxetine and indicated the potential for excretion-related DDIs when fluoxetine was coadministered with miltirone (Figures 4C, D).

**TABLE 2:** Pharmacokinetic parameters of fluoxetine in different groups after oral administration.

Parameters	Unit	Fluoxetine group	Fluoxetine + miltirone group
AUC_(0-t)_	μg/L*h	673.239 ± 367.221	1776.596 ± 384.091 **
AUC_(0-∞)_	μg/L*h	691.34 ± 359.347	1809.328 ± 377.821 **
MRT_(0-t)_	h	7.454 ± 1.869	11.135 ± 0.638**
MRT_(0-up)_	h	7.954 ± 1.433	12.075 ± 0.868**
T_1/2z_	h	2.798 ± 0.807	6.69 ± 2.551**
T_max_	h	6.833 ± 2.994	5.667 ± 1.506
CLz	L/h/kg	14.99 ± 8.688	4.563 ± 0.824 *
Vz	L/kg	65.888 ± 53.942	44.473 ± 19.134
C_max_	μg/L	85.35 ± 54.025	167.361 ± 38.513**

The values are presented as the means±SDs. n = 6. *p < 0.05 versus the control group, **p < 0.01 versus the control group.

**TABLE 3:** Pharmacokinetic parameters of norfluoxetine in different groups after oral administration.

Parameters	Unit	Fluoxetine group	Fluoxetine + miltirone group
AUC_(0-t)_	μg/L*h	13922.531 ± 5002.469	14679.733 ± 5587.335
AUC _(0-up)_	μg/L*h	16982.482 ± 3779.21	15071.949 ± 5990.102
MRT_(0-t)_	h	12.78 ± 1.977	15.313 ± 1.729
MRT_(0-up)_	h	18.475 ± 5.906	16.305 ± 1.972
T_1/2z_	h	9.041 ± 4.874	7.737 ± 1.157
T_max_	h	10 ± 2.191	10.667 ± 2.066
CLz	L/h/kg	0.492 ± 0.112	0.595 ± 0.208
Vz	L/kg	6.401 ± 3.38	6.491 ± 2.211
C_max_	μg/L	850.367 ± 199.914	721.717 ± 234.908

The values are presented as the means±SDs. n = 6. *p < 0.05 versus the control group.

### 3.4 Enzymatic studies of fluoxetine with CYP2D6 variants

**FIGURE 5:**
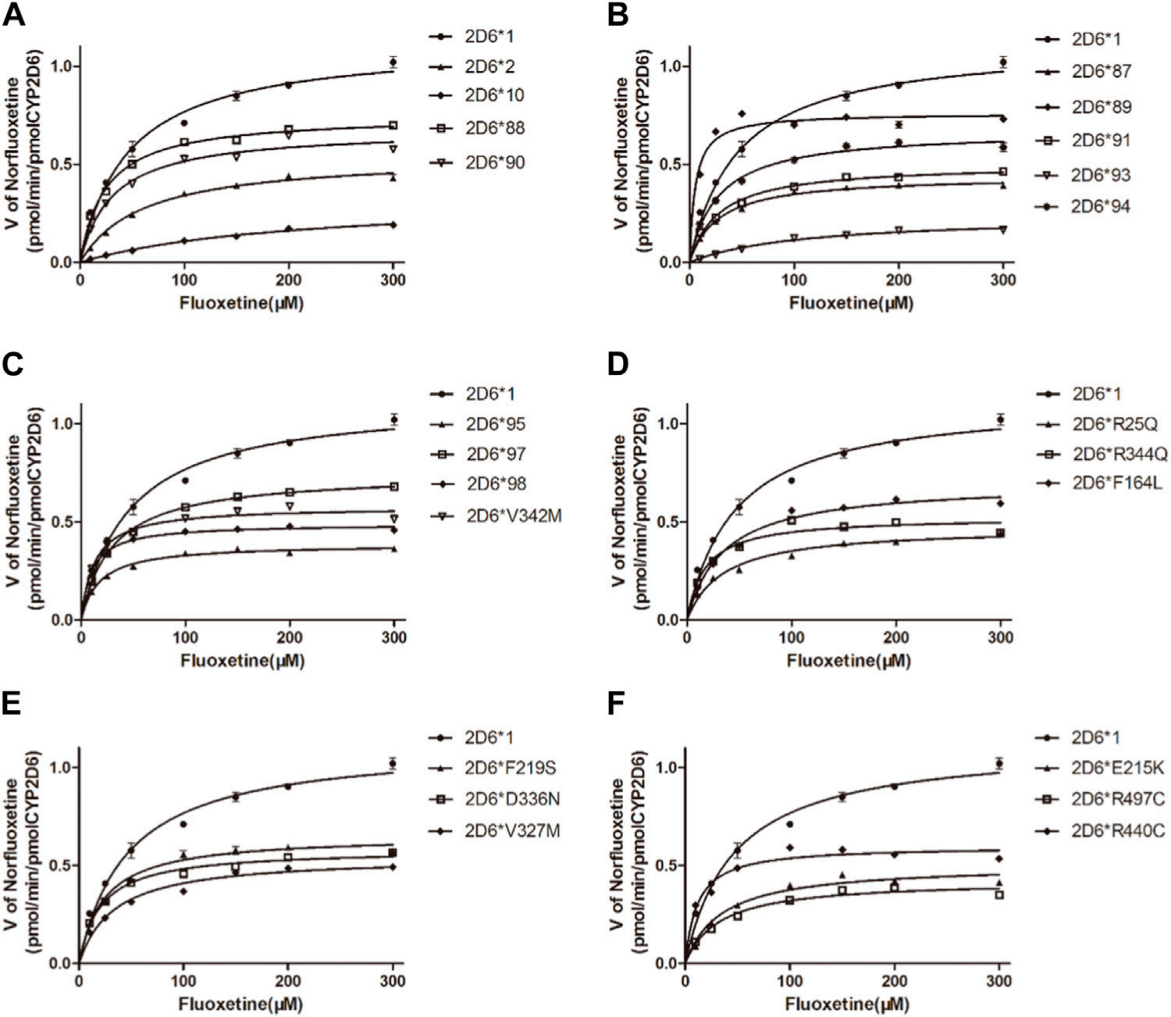
Michaelis–Menten curves of the enzymatic activity of the recombinant wild-type CYP2D6 protein (2D6*1) and 24 variants toward fluoxetine (e.g., 2D6*2, 2D6*10, 2D6*87, 2D6*88, 2D6*89, 2D6*90, 2D6*91, 2D6*93, 2D6*94, 2D6*95, 2D6*97, 2D6*98, 2D6*V342M, 2D6*R25Q, 2D6*R344Q, 2D6*F164L, 2D6*F219S, 2D6*D336N, 2D6*V327M, 2D6*E215K, 2D6*R497 and 2D6*R440C). The data are presented as the means ± SDs, *n*=3.

The Michaelis‒Menten curves are shown in Figure 5, and the corresponding K_m_ and V_max_ values are summarized in Table 4. As illustrated in Table 4, all the variants exhibited significantly reduced V_max_ values, and almost all exhibited significantly changed K_m_ values compared with those of wild-type protein. Therefore, the intrinsic clearance (V_max_/K_m_) values for fluoxetine demethylation were significantly altered in more than half of the tested allelic variants. CYP2D6*92 and *96 were inactive in terms of fluoxetine metabolism in this study and were thus classified as null alleles. The other 22 variants were classified into three types according to their relative clearance values compared to wild-type: the first type included 10 variants (CYP2D6*88/V104A, *90/K147R, *91/C161S, *94/D337G, *95/R388H, *98/H463D, *V327M, *F219S, *F164L and *D336N) that showed similar intrinsic clearance; 7 variants (CYP2D6*2, *93/T249P, *10, *87/A5V, *E215K, *R25Q and *R440C) exhibited significantly reduced V_max_/K_m_ values (6.62%–66.79%, *p* < 0.05) and were classified into the second type; and the remaining variants (CYP2D6*89/L142S, *R497, *97/F457L, *V342M and *R344Q) exhibited increased V_max_/K_m_ values of 578.83%, 212.57%, 201.62%, 188.50% and 125.07%, respectively, compared with wild type (*p* < 0.05).

**TABLE 4:** Kinetic parameters for the metabolic activities of recombinant wild-type and mutant CYP2D6 proteins with fluoxetine (n = 3).

Allelic protein	Allele frequency (%)	1000*V_max_ (pmol/min/pmol P450)	K_m_ (μM)	Clearance (V_max_/K_m_)	Relative clearance (/CYP2D6*1) (%)
CYP2D6*89/L142S	0.023	760.3 ± 14.44^##^	5.44 ± 0.84^#^	139.96 ± 4.57^##^	578.83^##^
CYP2D6*R497C	0.023	598.6 ± 13.99^##^	11.67 ± 1.547^#^	51.40 ± 3.70^#^	212.57^#^
CYP2D6*97/F457L	0.047	489.8 ± 4.334^##^	10.06 ± 0.54^#^	48.75 ± 2.94^#^	201.62^#^
CYP2D6*V342M	0.023	578.5 ± 10.89^##^	12.71 ± 1.31^#^	45.58 ± 2.79^##^	188.50^##^
CYP2D6*R344Q	0.023	524.9 ± 15.02^##^	17.36 ± 2.42^#^	30.24 ± 0.99^#^	125.07^#^
CYP2D6*88/V104A	0.094	748.3 ± 10.28^#^	24.46 ± 1.44^#^	30.67 ± 1.99	126.84
CYP2D6*V327M	0.023	581.8 ± 8.32^##^	20.72 ± 1.35^#^	28.12 ± 2.01	116.28
CYP2D6*F219S	0.023	653.2 ± 13.09^##^	23.74 ± 2.07^#^	27.52 ± 0.19	113.81
CYP2D6*98/H463D	0.023	746.9 ± 11.15^#^	30.05 ± 1.79	25.18 ± 3.05	104.14
CYP2D6*94/D337G	0.164	670.6 ± 13.2^##^	27.33 ± 2.22^#^	24.56 ± 1.86	101.58
CYP2D6*1	26.56	1122.0 ± 35.03	46.3 ± 4.90	24.18 ± 2.79	100.00
CYP2D6*95/R388H	0.047	386.2 ± 6.302^##^	17.49 ± 1.39^#^	22.09 ± 1.25	91.37
CYP2D6*90/K147R	0.047	675.6 ± 18^##^	31.16 ± 3.27	21.68 ± 0.72	89.66
CYP2D6*F164L	0.023	699.8 ± 16.84^##^	35.04 ± 3.173	19.98 ± 0.58	82.61
CYP2D6*91/C161S	0.023	502.3 ± 7.79^##^	28.51 ± 1.80	17.63 ± 0.78	72.93
CYP2D6*D336N	0.023	545.9 ± 14.27^##^	33.4 ± 3.35	16.36 ± 0.51	67.68
CYP2D6*87/A5V	0.023	442.2 ± 6.452^##^	27.37 ± 1.65^#^	16.15 ± 0.32^#^	66.79^#^
CYP2D6*E215K	0.047	504.7 ± 16.73^##^	34.47 ± 4.33^#^	14.66 ± 1.07^#^	60.64^#^
CYP2D6*R25Q	0.023	472.7 ± 11.56^##^	34.48 ± 3.19	13.71 ± 0.26^#^	56.70^#^
CYP2D6*R440C	0.023	424.8 ± 12.09^##^	32.64 ± 3.59	13.02 ± 0.32^#^	53.83^#^
CYP2D6*2	10.34	545.4.±14.82^##^	59.99 ± 4.99	9.09 ± 0.37^#^	37.60^#^
CYP2D6*93/T249P	0.023	237.9 ± 11.5^##^	108.5 ± 12.64^#^	2.20 ± 0.13^##^	9.08^##^
CYP2D6*10	42.86	328.7 ± 17.98^##^	205.6 ± 21.1^##^	1.60 ± 0.05^##^	6.62^##^
CYP2D6*92	0.023	N.D.	N.D.	N.D.	N.D.
CYP2D6*96/Q424X	0.074	N.D.	N.D.	N.D.	N.D.

N.D., indicates that the metabolite was not detected; therefore, kinetic parameters for the fluoxetine activities of some recombinant mutant variants cannot be calculated. # represents *p* < 0 .05 vs. wild-type. ## represents *p* < 0.01 vs. wild-type.

### 3.5 Enzymatic studies with diverse antidepressants

As shown in Table 5, to understand the differences in the metabolism of diverse antidepressants by reported CYP2D6 variants, we compared the relative clearance values of venlafaxine, citalopram, olanzapine, and fluvoxamine reported with those of fluoxetine in this study (Hu et al., 2016; Ye et al., 2022; Zhan et al., 2016; Zhou et al., 2016).

**TABLE 5:** Enzymatic activity of the wild-type and 24 CYP2D6 variant proteins toward venlafaxine, citalopram, olanzapine, fluvoxamine and fluoxetine metabolism (n = 3).

Allelic protein	V_max_/K_m_ (% of the wild-type)				
	Venlafaxine Zhan et al. (2016)	Citalopram Hu et al. (2016)	Olanzapine Zhou et al. (2016)	Fluvoxamine Ye et al. (2022)	Fluoxetine
CYP2D6*1	100.00	100.00	100.00	100.00	100.00
CYP2D6*2	28.3^#^	43.22^#^	39.7^#^	55.254	37.60^#^
CYP2D6*10	2.9^#^	47.44^#^	5.2^#^	101.865	6.62^#^
CYP2D6*87/A5V	38.3^#^	39.38^#^	26.0^#^	393.295^#^	66.79^#^
CYP2D6*88/V104A	49.4^#^	65.96^#^	78.9^#^	176.140^#^	126.84
CYP2D6*89/L142S	71.1^#^	62.99^#^	137.8^#^	151.434	578.83^#^
CYP2D6*90/K147R	75.1^#^	65.34^#^	75.8^#^	179.493	89.66
CYP2D6*91/C161S	37.9^#^	43.77^#^	75.7^#^	38.336	72.93
CYP2D6*92	N.D	N.D	N.D.	N.D	N.D.
CYP2D6*93/T249P	0.2^#^	49.93^#^	17.7^#^	48.790	9.08^#^
CYP2D6*94/D337G	71.9^#^	109.76	81.9^#^	153.626^#^	101.58
CYP2D6*95/R388H	47.7^#^	75.21^#^	68.4^#^	126.083	91.37
CYP2D6*96/Q424X	N.D.	N.D.	N.D.	N.D.	N.D.
CYP2D6*97/F457L	44.2^#^	82.21^#^	75.3^#^	96.463	201.62^#^
CYP2D6*98/H463D	56.6^#^	70.82^#^	115.7^#^	—	104.14
CYP2D6*V342M	84.4^#^	129.33^#^	82.5^#^	394.310	188.50^#^
CYP2D6*R25Q	20.3^#^	60.63^#^	48.5^#^	—	56.70^#^
CYP2D6*F164L	65.6^#^	47.92^#^	61.4^#^	218.589^#^	82.61
CYP2D6*R344Q	43.6^#^	41.88^#^	72.9^#^	123.621	125.07^#^
CYP2D6*F219S	39.3^#^	121.05	60.0^#^	71.168	113.81
CYP2D6*D336N	46.5^#^	68.45^#^	104.5	157.504	67.68
CYP2D6*V327M	22.4^#^	91.13	48.1^#^	157.504	116.28
CYP2D6*E215K	2.4^#^	106.45	80.5^#^	74.351	60.64^#^
CYP2D6*R497C	70.1^#^	66.69^#^	32.0^#^	331.993	212.57^#^
CYP2D6*R440C	28.2^#^	38.46^#^	28.2^#^	317.234^#^	53.83^#^

N.D., indicates that the metabolite was not detected; therefore, kinetic values for the fluoxetine activities of some recombinant mutant variants cannot be calculated. — indicates that some variants were not identified in previous research. # represents *p* < 0.05 vs. wild-type.

## 4 Discussion

Some major tanshinones isolated from Salvia miltiorrhiza (Danshen in Chinese) can inhibit human and rat CYP450 enzyme-mediated metabolism of model probe substrates and have the potential to cause herb‒drug interactions (Zhou et al., 2013). Miltirone, an active tanshinone compound isolated from Danshen, has been reported to have strong antioxidative and anxiolytic effects (Chang et al., 1991; Zhou et al., 2013). Previous studies have shown that miltirone inhibits CYP1A2, CYP2C9, CYP2D6, CYP2C19 and CYP3A4 in pooled human liver microsomes (Hu et al., 2015; Zhou et al., 2013). Our *in vitro* screening results and *in vivo* data both indicated that miltirone inhibited the CYP450 enzyme-mediated metabolism of fluoxetine in rats, which might increase the risk of adverse effects.

CYP450 inhibition by drugs is usually reversible but is sometimes irreversible. For example, mibefradil is a time-dependent inhibitor that irreversibly inhibits CYP3A4, which can lead to a significant increase in the blood concentration of coprescribed drugs, resulting in toxicity and even death (Foti et al., 2011). Like the irreversible covalent inhibitor sinomenine we discussed in a prior report, miltirone also possesses a cyclic Michael acceptor (i.e., an α,β-unsaturated ketone structure) that is potentially reactive with the nucleophilic residues of target proteins (Chen et al., 2021). Therefore, we hypothesized that miltirone could irreversibly bind to cytochrome P450 enzymes, resulting in structural changes in these enzymes and their inactivation. As summarized in Figure 1C, the inhibitory effect of miltirone on the metabolism of fluoxetine in RLMs with an IC_50_ at a low micromolar concentration was used to determine the reversible inhibitory effect of miltirone on CYP450 enzymes. However, this method is not an ideal potency metric for irreversible time-dependent inhibitors (TDIs), which can be assessed by performing IC_50_ shift assays (Perloff et al., 2009). Overall, the observed IC_50_ shift (0.55-fold) in this study suggested that miltirone is likely a reversible CYP inhibitor in RLMs (Figure 1D).

In addition to metabolism-based DDIs, the CYP450 pharmacogenetic phenotype is another main reason for the variability of drug responses. To date, we have systematically analyzed the enzymatic characteristics of 39 CYP2C9 isoforms and 31 CYP2C19 isoforms toward fluoxetine and determined that more than 75% of the variants exhibited significantly decreased enzymatic activity (Ji et al., 2015; Fang et al., 2017). As shown in Table 4, the K_m_ for fluoxetine of recombinant wild-type CYP2D6 was 46.3 μM, which is similar to that of CYP2C9 (31.7 μM) but lower than that of CYP2C19 (97.6 μM), indicating that fluoxetine has a greater affinity for CYP2D6 than for CYP2C19. Previous studies have also indicated that CYP2D6 has an important rol in the formation of (R)-NFLX (accounting for ∼40%), except for CYP2C9, whereas (S)-FLX N-demethylation (the formation of S-NFLX) is correlated with the catalytic activity of CYP2D6 only (Fuller et al., 1992; Fjordside et al., 1999).

Patients with different CYP2D6 variants have interindividual drug response variability and can be classified into four types: poor metabolizers (PMs), intermediate metabolizers (IMs), extensive metabolizers (EMs) and ultrarapid metabolizers (UMs) (Aly et al., 2022). Some articles have shown that CYP2D6 PMs are more likely to experience adverse reactions and even death, when they take normal doses of fluoxetine (Haufroid et al., 2015; Sallee et al., 2000; Wang et al., 2014). Thus, it is essential to evaluate the roles of different CYP2D6 variants in the metabolism of fluoxetine and design individualized therapies. For instance, a total of 9 allelic variants exhibited decreased clearance rates compared to the wild-type, as summarized in Table 4, due to an increased in the K_m_ and a decreased in the V_max_. Among these, four allelic isoforms (CYP2D6*10, CYP2D6*92, CYP2D6*93/T249P and CYP2D6*96) retained less than 10% of the metabolic activity of the wild-type. The allele frequency (%) of CYP2D6*10, CYP2D6*92, CYP2D6*93/T249P and CYP2D6*96 in our study were 0.023%, 42.86%, 0.023% and 0.074%, respectively. Therefore, it is expected that nearly half of the population in China may have any one of the four isomers with less than 10% activity towards fluoxetine metabolism and greater care should be taken when using fluoxetine in patients with those mutants. In contrast, five new types exhibited higher clearance rates than the wild type, which might contributes towards why 30%–40% of patients do not respond to fluoxetine treatment (Blazquez et al., 2012). It would be interesting to determine the clinical importance of these novel CYP2D6 allele variants on fluoxetine metabolism in patients.

Accurate prediction of the CYP2D6 phenotype from genotype information is important to support safe and efficacious pharmacotherapy with different CYP2D6 substrates (Frederiksen et al., 2023). As shown in Table 5, the relative clearance values of venlafaxine by all CYP2D6 variants decreased significantly compared with that of the wild-type, which is different from the results for olanzapine and citalopram reported previously or for fluoxetine in this study (Hu et al., 2016; Zhan et al., 2016; Zhou et al., 2016). Furthermore, more than 8 CYP2D6 variants exhibited slightly increased enzymatic activity (>10%) toward fluoxetine and fluvoxamine among these variants (Ye et al., 2022), but only 2 variants exhibited slightly increased enzymatic activity toward olanzapine (CYP2D6*V342M, CYP2D6*F219S) and citalopram (CYP2D6*89/L142S, CYP2D6*98/H463D). Similar to the results for olanzapine and citalopram, 5 variants (CYP2D6*10, CYP2D6*87/A5V, CYP2D6*90/K147R, CYP2D6*F164L and CYP2D6*R440C) exhibited notably decreased enzymatic activity (>10%) for fluoxetine but increased metabolic activity for fluvoxamine. In summary, almost all CYP2D6 variants exhibited different activities against different substrates compared to the wild-type, except for CYP2D6*2, CYP2D6*91/C161S and CYP2D6*93/T249P. These results suggest that amino acid substitutions at these sites can lead to substrate-dependent intrinsic enzymatic differences.

In summary, our results highlight that miltirone contributes to the inhibition of fluoxetine metabolism in RLMs and rats. This study provides valuable information regarding the interactions of fluoxetine with miltirone. However, the DDI of fluoxetine and miltirone in the human body remains to be confirmed in further studies. Moreover, the present study provides the first comprehensive analysis of the demethylation activities of CYP2D6*1, CYP2D6*2, CYP2D6*10 and 22 new alleles in the metabolism of fluoxetine *in vitro*. Information about the metabolism of fluoxetine by different variants may provide an important foundation for future clinical studies.

## Data Availability

The raw data supporting the conclusion of this article will be made available by the authors, without undue reservation.
